# A Novel Ferroptosis-Related Biomarker Signature to Predict Overall Survival of Esophageal Squamous Cell Carcinoma

**DOI:** 10.3389/fmolb.2021.675193

**Published:** 2021-07-05

**Authors:** Jiahang Song, Yanhu Liu, Xiang Guan, Xun Zhang, Wenda Yu, Qingguo Li

**Affiliations:** ^1^ Cardiovascular Center, The Second Affiliated Hospital of Nanjing Medical University, Nanjing, China; ^2^ Department of Radiation Oncology, The First Affiliated Hospital of Nanjing Medical University, Nanjing, China; ^3^ Department of Cardiovascular Surgery, The Affiliated Hospital of Qinghai University, Xining, China

**Keywords:** esophageal squamous cell carcinoma, ferroptosis, prognosis, gene signature, TCGA, GEO

## Abstract

Esophageal squamous cell carcinoma (ESCC) accounts for the main esophageal cancer (ESCA) type, which is also associated with the greatest malignant grade and low survival rates worldwide. Ferroptosis is recently discovered as a kind of programmed cell death, which is indicated in various reports to be involved in the regulation of tumor biological behaviors. This work focused on the comprehensive evaluation of the association between ferroptosis-related gene (FRG) expression profiles and prognosis in ESCC patients based on The *Cancer* Genome Atlas (TCGA) and Gene Expression Omnibus (GEO). ALOX12, ALOX12B, ANGPTL7, DRD4, MAPK9, SLC38A1, and ZNF419 were selected to develop a novel ferroptosis-related gene signature for GEO and TCGA cohorts. The prognostic risk model exactly classified patients who had diverse survival outcomes. In addition, this study identified the ferroptosis-related signature as a factor to independently predict the risk of ESCC. Thereafter, we also constructed the prognosis nomogram by incorporating clinical factors and risk score, and the calibration plots illustrated good prognostic performance. Moreover, the association of the risk score with immune checkpoints was observed. Collectively, the proposed ferroptosis-related gene signature in our study is effective and has a potential clinical application to predict the prognosis of ESCC.

## Introduction

Esophageal cancer (ESCA), a global malignancy, ranks sixth and eighth in terms of tumor-related mortality and morbidity of all tumors, respectively. ESCA is associated with a dismal prognostic outcome, and its five-year survival rate has been reported to be 15–25%. Esophageal squamous cell carcinoma (ESCC) accounts for a major ESCA subtype, which is predominant in eastern Asia (Matsushima et al., 2010). The poor outcome of ESCC is associated with its insidious initial symptoms, susceptibility to metastasis, resistance to radiotherapy, and tumor recurrence (Pennathur et al., 2013). Over the past few years, multidisciplinary and surgical treatments have been developed, but the median survival of ESCC cases is only 10 months (Wang et al., 2020a). Moreover, considering the limited prediction of prognosis for ESCC patients, there is an urgent need for the exploration of novel biomarkers.

Ferroptosis, the novel regulated cell death (RCD) type that is different from necrosis, apoptosis, and autophagy, is featured by lipid hydroperoxide accumulation till the lethal dose (Dixon et al., 2012). As revealed by more and more studies, ferroptosis exerts an important part in tumor progression and treatment (Stockwell et al., 2017; Shen et al., 2018; Gan, 2019). Besides, various tumor types such as adrenocortical carcinoma, hepatocellular carcinoma, and ovarian cancer have been demonstrated to be sensitive to ferroptosis (Yang et al., 2014; Belavgeni et al., 2019; Carbone and Melino, 2019). Numerous reports have indicated that ferroptosis-related genes (FRGs) are involved in the regulation of tumor initiation and progression (Junttila and Evan, 2009; Arrigo and Gibert, 2012; Liu et al., 2018; Enz et al., 2019). ALOX12 exhibits a context-dependent role in mediating lipid peroxidation, resulting in PUFA oxidation which promotes cell ferroptosis. An outstanding report was performed by Chu et al., who uncovered that ALOX12 is essential for p53-mediated tumor ferroptosis through the ACSL4-independent pathway (Chu et al., 2019). Recent studies confirmed that ANGPTL7 and DRD4 were inhibited by ferroptotic erastin, indicating the potential role of being ferroptosis markers (Yang et al., 2014; Wang et al., 2016). Gao et al. proved that repression of glutamine metabolism could reduce cell ferroptosis, which revealed a novel function of SLC38A1 in regulated cell death (Gao et al., 2015). However, the relationship between these FRGs and prognostic outcomes for ESCC cases remains to be further examined.

This study downloaded ESCC patient samples and corresponding clinical information from GEO and TCGA public databases. Afterward, we successfully established the prognosis risk signature that incorporated seven FRGs based on the GEO training set and validated it in the GEO test set, entire GEO set, and TCGA dataset. Ultimately, we initially explored the oncogenic effect of SLC38A1 through *in vitro* studies. This work develops a novel FRG prognostic signature to improve the prediction of the clinical outcomes of ESCC patients.

## Materials and Methods

### Data Collection

Expression RNA-seq data together with associated clinical data from ESCC cases were acquired from The *Cancer* Genome Atlas (TCGA) data portal (https://tcga-data.nci.nih.gov/tcga/) and the Gene Expression Omnibus (GEO) (http://www.ncbi.nlm.nih.gov/geo/) database, which defined the entire GSE53625 set (*n* = 179) and TCGA set (*n* = 81), respectively. A total of 255 FRGs were extracted from the FerrDb website (http://www.zhounan.org/ferrdb).

### Identification of Ferroptosis-Related Gene Prognostic Signature

Firstly, the entire GSE53625 set (*n* = 179) was randomized as the training set together with the internal test set in the 1:1 ratio. Then, we performed univariate Cox regression analysis for identifying prognostic FRGs (*p* < 0.05) in the training cohort. To remove the potential overfitting genes, the “glmnet” package was adopted for least absolute shrinkage and selection operator (LASSO) regression. At last, the optimal prognosis model based on FRGs was constructed by multivariate Cox regression. To be specific, we determined the risk score for ESCC cases by the following formula: risk score = (Gene 1 expression × coefficient) + (Gene 2 expression × coefficient) + … + (Gene n expression × coefficient). Meanwhile, the cases were separated into high- or low-risk groups based on the median score. In addition, the test set, entire set, and TCGA set were used to validate our signature.

### Nomogram Establishment and Validation

For predicting the clinical outcomes of ESCC patients, we utilized the R package “rms” to construct the nomogram which incorporated clinical factors and risk signature. Additionally, the nomogram performance and prediction accuracy were determined to plot the calibration curves.

### Gene Set Enrichment Analysis

GSEA was employed to detect biological functions as well as related signaling pathways in the high-risk group. The expression of genes in both the high- and low-risk groups, together with the collection of Hallmark and KEGG gene sets in Molecular Signatures Database v7.1, was analyzed by GSEA software. Gene sets conforming to | NES |> 1 and NOM *p* < 0.05 were deemed significant based on the GSEA User Guide.

### Validation of Protein Expressions of Signature Genes by the HPA Database

Immunohistochemistry (IHC) helps to uncover relative protein distribution and expression according to particular binding of antigens with antibodies. IHC was conducted to determine the prognostic FRG expression in ESCC and non-carcinoma samples from the Human Protein Atlas (HPA, https://www.proteinatlas.org/) database at the protein level.

### Cell Culture and Cell Transfection

ESCC cell lines (Eca109 and KYSE-150), together with the normal human esophageal epithelial cells (HEECs), were cultivated within the RPMI‐1640 medium containing 10% fetal bovine serum (FBS, Gibco Company) and 10% streptomycin–penicillin (Sigma‐Aldrich) and incubated in an incubator under 37°C and 5% CO_2_ conditions. In addition, si‐SLC38A1 and siRNA negative control (si‐NC) were prepared via Ribobio (Guangzhou, China). The sense sequence of si-SLC38A1 was 5′-GUU​ACC​UUC​AAU​UCA​AAG​ATT-3′. Later, Lipofectamine 3000 reagent (Invitrogen) was employed to transfect siRNAs to specific cells in line with specific protocols. After transfection for 48 h, we harvested cells to conduct later experiments.

### Quantitative Reverse Transcription Polymerase Chain Reaction

We utilized Trizol reagent (Vazyme Biotech, Nanjing, China) to isolate the total cellular RNA from ESCC cells. All extraction steps were performed in line with specific protocols. The BioSpec-nano spectrophotometer (Shimadzu, Japan) was used to measure the extracted RNA content. We deemed RNA samples that had the A260/A280 ratio of 1.8–2 as suitable samples. We then reverse transcribed the RNA using Prime Script RT Master Mix reagent (Takara Bio, Dalian, China) for obtaining cDNA. The PCR system was prepared to utilize TB Green^®^ Premix Ex Taq™ (Takara Bio, Dalian, China). We performed qRT-PCR on the Applied Biosystems StepOnePlus real-time PCR system (Thermo Fisher Scientific). In addition, the 2^−ΔΔCT^ approach was applied in calculating the relative gene level. The SLC38A1 level was analyzed by the following primers: 5′-GAT​GGG​TGA​TGG​TGA​TAG​GG-3′ (forward) and 5′-TAC​TGG​TCT​AGG​GGC​CAC​AC-3′ (reverse). GAPDH was used as a reference gene.

### Western Blot Analysis

Western blot analysis was conducted for determining SLC38A1 and GAPDH levels. The SLC38A1 (#36057, 1:1,000) and GAPDH (#5174, 1:1,000) antibodies were provided by Cell Signaling Technology (CST, Danvers, MA, United States).

### Cell Counting Kit-8 Assay

We used the CCK-8 kit (Beyotime, Shanghai, China) for determining cell proliferation following specific protocols. The cells (2000/well) were inoculated into the 96-well plates and cultured within RPMI-1640 that contained 10% FBS. At a fixed time of day, we added CCK-8 solution into each well to incubate cells under 37°C for additional 2 h. The absorbance (OD) value was detected at 450 nm.

### Colony Formation Assay

The cells (250/well) after transfection were inoculated to six-well plates in the colony formation assay and cultured within the RPMI-1640 medium that contained 10% FBS for a period of 10 days. Later, 1% formaldehyde was used to fix the growing colonies, whereas 1% crystal violet was utilized to stain the colonies. After taking images, we counted the colony number.

### Transwell Assay

The Transwell chamber (pore size, 8 μm; Corning Costar Corp, United States) was utilized to examine cell migration. In brief, after suspending the stably transfected ESCC cells into the serum-free RPMI-1640 medium (200 μL), the upper chamber was loaded with cell suspension. Afterward, the RPMI-1640 medium (500 μL) that contained 10% FBS was placed into the lower chamber, followed by 24 h of cell incubation under 37°C. Later, 1% crystal violet was used to stain cells for 20 min, and then cotton swabs were used to remove cells on the upper membrane surface. A microscope (Olympus) was used to take photographs of cells on the bottom membrane surface, and four random fields were utilized to count the migration cells.

### Statistical Analysis

R software (3.6.3) and GraphPad (8.0) were employed for all statistical data analyses. The log-rank test and Kaplan–Meier analysis were adopted for evaluating different OS between high- and low-risk groups. Besides, univariate and multivariate Cox regression was applied in identifying those independent factors for predicting prognosis. Time-dependent receiver operating characteristic (ROC) curves were used to evaluate our risk model for its prediction performance. A difference of *p* < 0.05 indicated statistical significance.

## Results

### Construction and Verification of the Ferroptosis-Related Gene Prognostic Signature

**FIGURE 1:**
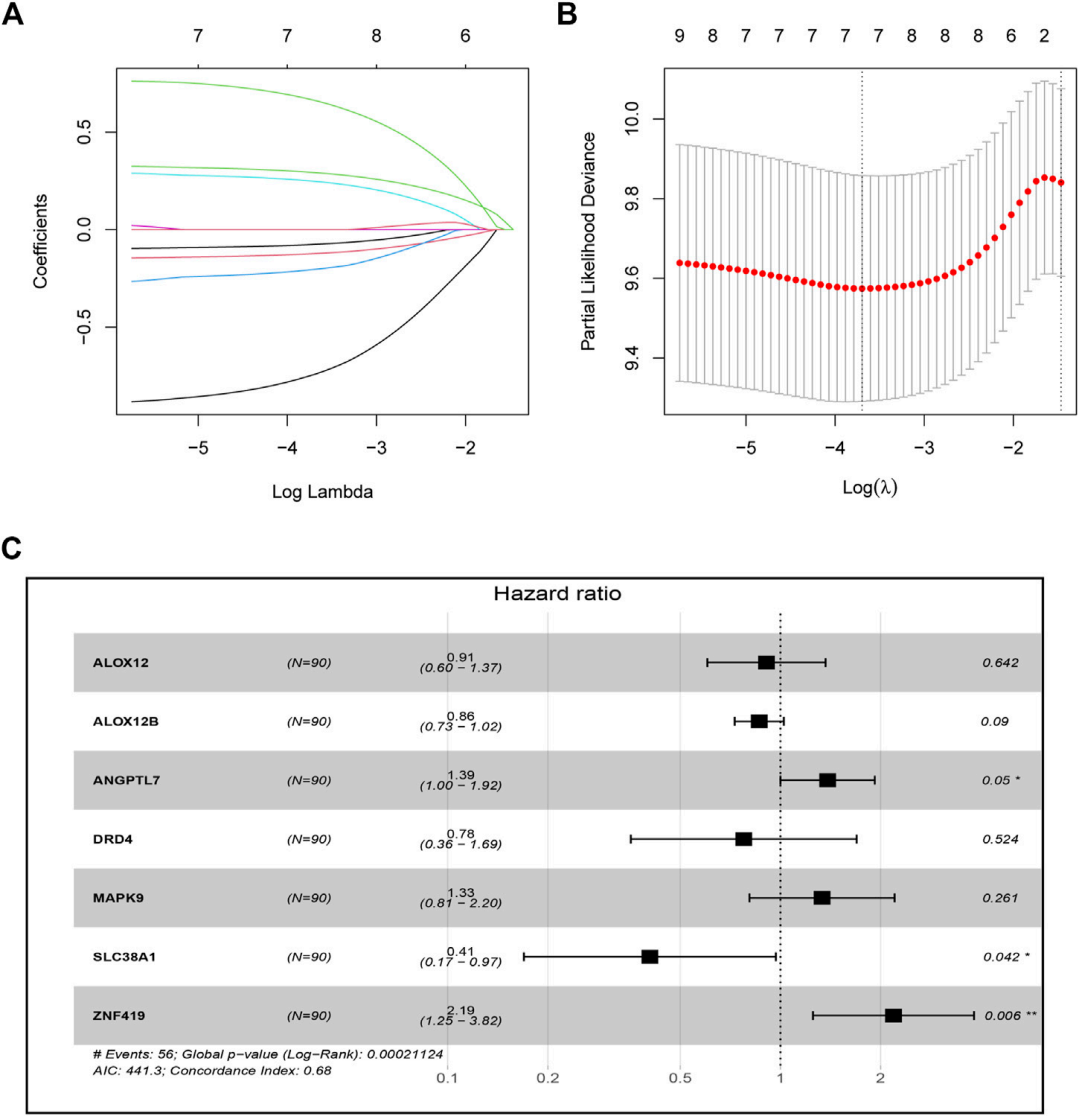
Construction of the seven-ferroptosis-gene signature. **(A)** Cross-validation for tuning parameter screening upon LASSO regression analysis. **(B)** LASSO coefficient profiles for those intersected genes. **(C)** Forest plot of hazard ratios exhibiting the prognostic worth of seven FRGs.

**FIGURE 2:**
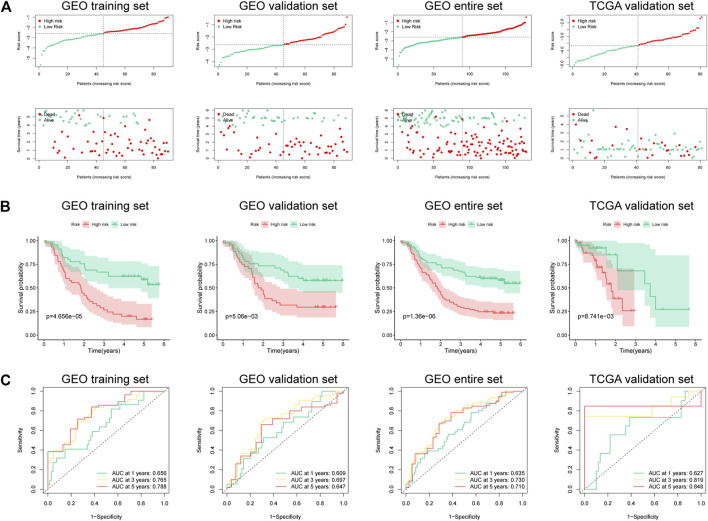
Risk score of the prognostic signature that comprises seven FRGs for OS in four cohorts. **(A)** Risk score distribution and survival status of high- and low-risk patients. **(B)** Kaplan–Meier analysis on high- and low-risk patients. **(C)** Time-dependent ROC curve analyses on the GEO training set, GEO validation set, entire GEO set, and TCGA validation set.

A total of 179 ESCC patients from GSE53625 were randomized in a 1:1 ratio into a training cohort (90 samples) and an internal validation cohort (89 samples). LASSO regression and multivariate Cox regression were performed in the training set to identify seven ferroptosis-related genes (ALOX12, ALOX12B, ANGPTL7, DRD4, MAPK9, SLC38A1, and ZNF419) for constructing a novel prognostic signature (Figure 1). The formula is shown as follows: Risk score = [ALOX12 expression× (−0.097)] + [ALOX12B expression× (−0.147)] + [ANGPTL7 expression× (0.326)] + [DRD4 expression × (−0.254)] + [MAPK9 expression × (0.288)] + [SLC38A1 expression× (−0.904)] + [ZNF419 expression × (0.782)]. We classified the ESCC cases into low- and high-risk groups based on the median risk score. The predictive performance of our seven-FRG–based risk model to predict patient OS can be observed in Figure 2A. As suggested through the Kaplan–Meier curve plotted according to the log-rank test, high-risk patients had poor OS compared with low-risk patients (*p* < 0.05, Figure 2B). For evaluating the credibility of our constructed model in predicting prognosis, we conducted ROC curve analysis. According to Figure 2C, area under the curve (AUC) values for the one-, three-, and five-year survival were determined to be 0.656, 0.765, and 0.788, respectively, for the GEO training set. The same analysis was conducted in the GEO validation cohort, and the AUC values for one-, three-, and five-year survival were 0.609, 0.697, and 0.647, respectively (Figure 2C). Moreover, we observed similar results in TCGA and the entire GEO sets, which proved the strong predictive potential of our risk model (Figure 2).

### Subgroup Analysis for the Ferroptosis-Related Gene Prognostic Signature

**FIGURE 3:**
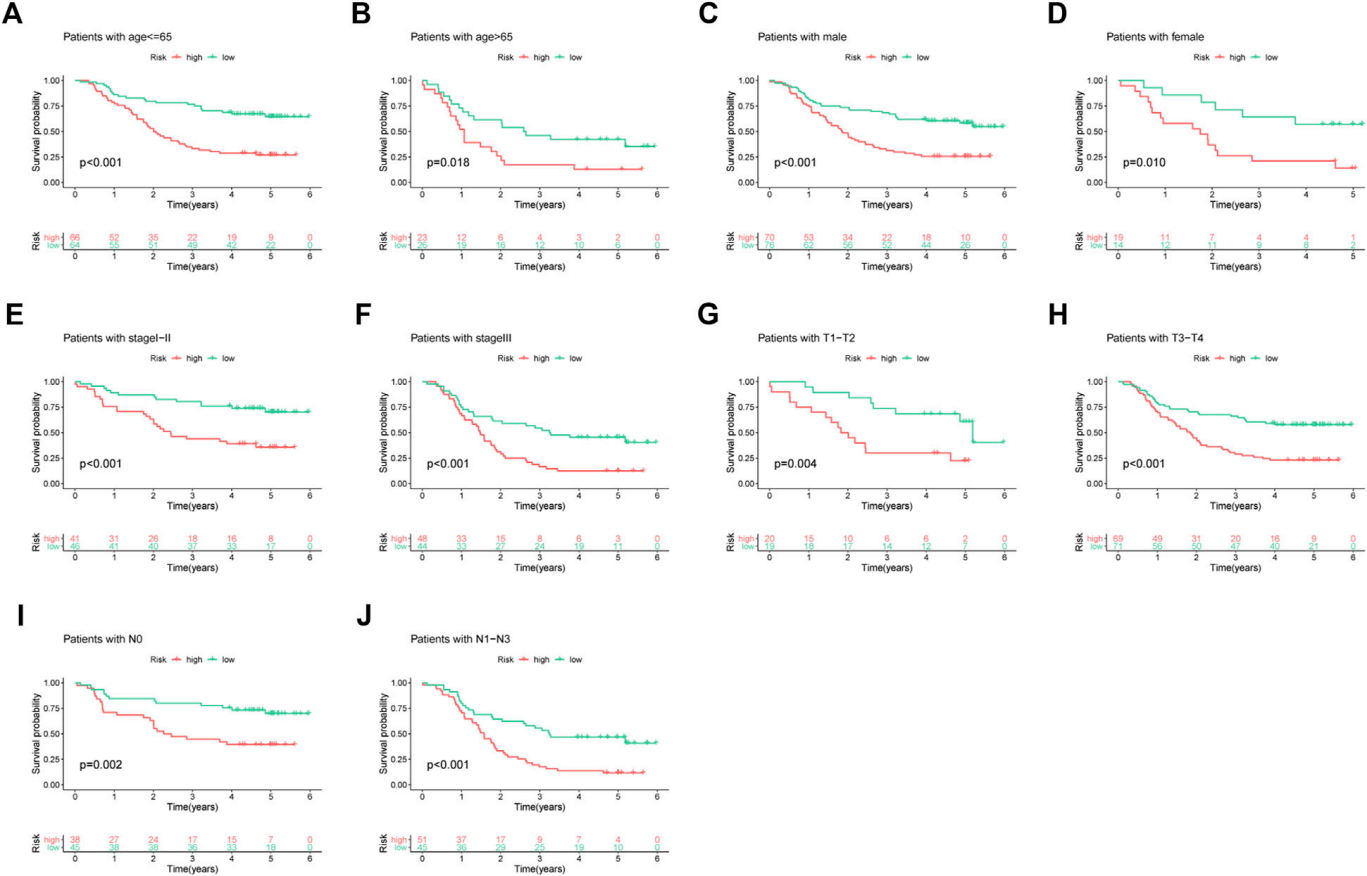
Subgroup analyses based on different clinical features of ESCC cases: **(A)** age ≤65, **(B)** age >65, **(C)** male, **(D)** female, **(E)** stage I-II, **(F)** stage III, **(G)** T1-T2, **(H)** T3-T4, **(I)** N0, and **(J)** N1–N3.

This study determined the predictive performance of the prognostic signature for OS of patients who had diverse clinical parameters. As a result, subgroups were categorized according to age (≤65 vs. >65 years), gender (male vs. female), clinical stage (I-II vs. III), T stage (T1 + T2 vs. T3 + T4), and N stage (N0 vs. N1–N3). Based on age, gender, clinical stage, T stage, and N stage, high-risk patients had markedly poor five-year OS rates compared with low-risk patients (Figure 3).

### Prognostic Nomogram Establishment and Validation

**FIGURE 4:**
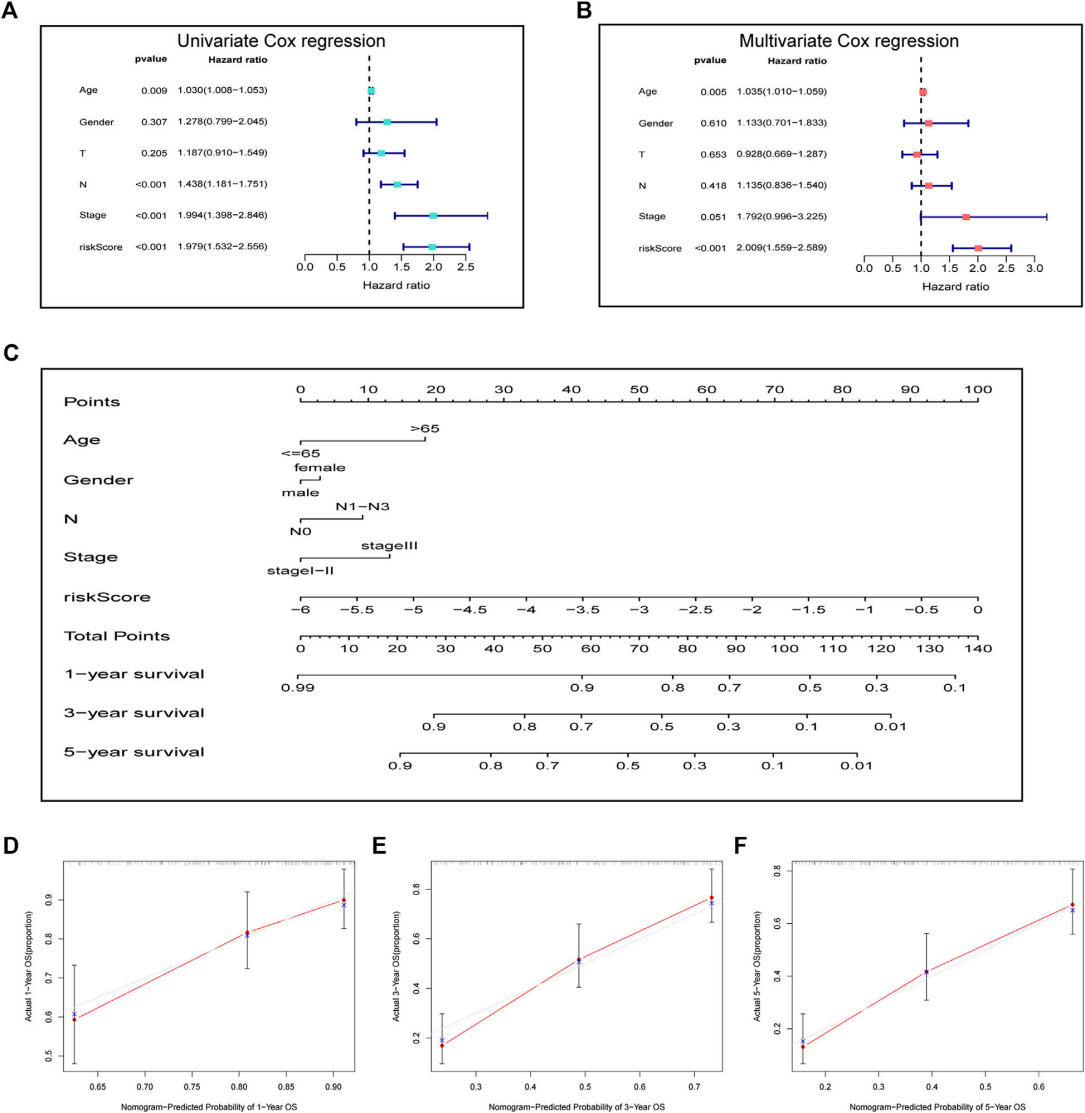
Prognostic signature in combination with clinical parameters for predicting prognostic outcomes for ESCC cases. **(A)** Univariate analysis and **(B)** multivariate analysis containing the risk score and clinical factors. **(C)** Nomogram for predicting one-, three-, and five-year OS. **(D–F)** Calibration curves of nomogram on consistency between predicted and observed one-, three-, and five-year survival.

For investigating the possibility of using the as-constructed prognosis nomogram as the factor to independently predict the prognosis for ESCC cases, univariate together with multivariate Cox regression was carried out. As revealed by univariate analysis, age (*p* = 0.009), risk score (*p* < 0.001), N stage (*p* < 0.001), and clinical stage (*p* < 0.001) predicted the dismal OS (Figure 4A). In addition, according to multivariate Cox regression, the risk score (HR = 2.009, 95% CI = 1.559–2.589, *p* < 0.001) and age (HR = 1.034, 95% CI = 1.010–1.059, *p* = 0.005) were identified as the independent prognostic factors that predicted the poor OS for ESCC cases (Figure 4B). Subsequently, we incorporated the risk score and other clinicopathologic characteristics to establish a novel nomogram to predict the one-, three-, and five-year OS rates of ESCC patients (Figure 4C). Every individual patient would acquire a corresponding score, and a higher total point demonstrates a poorer outcome for the patient. Moreover, the one-, three-, and five-year survival calibration curves well fitted our constructed nomogram in the GEO entire cohort (Figures 4D–F).

### Gene Set Enrichment Analysis With the Ferroptosis-Related Gene Prognostic Signature

**FIGURE 5:**
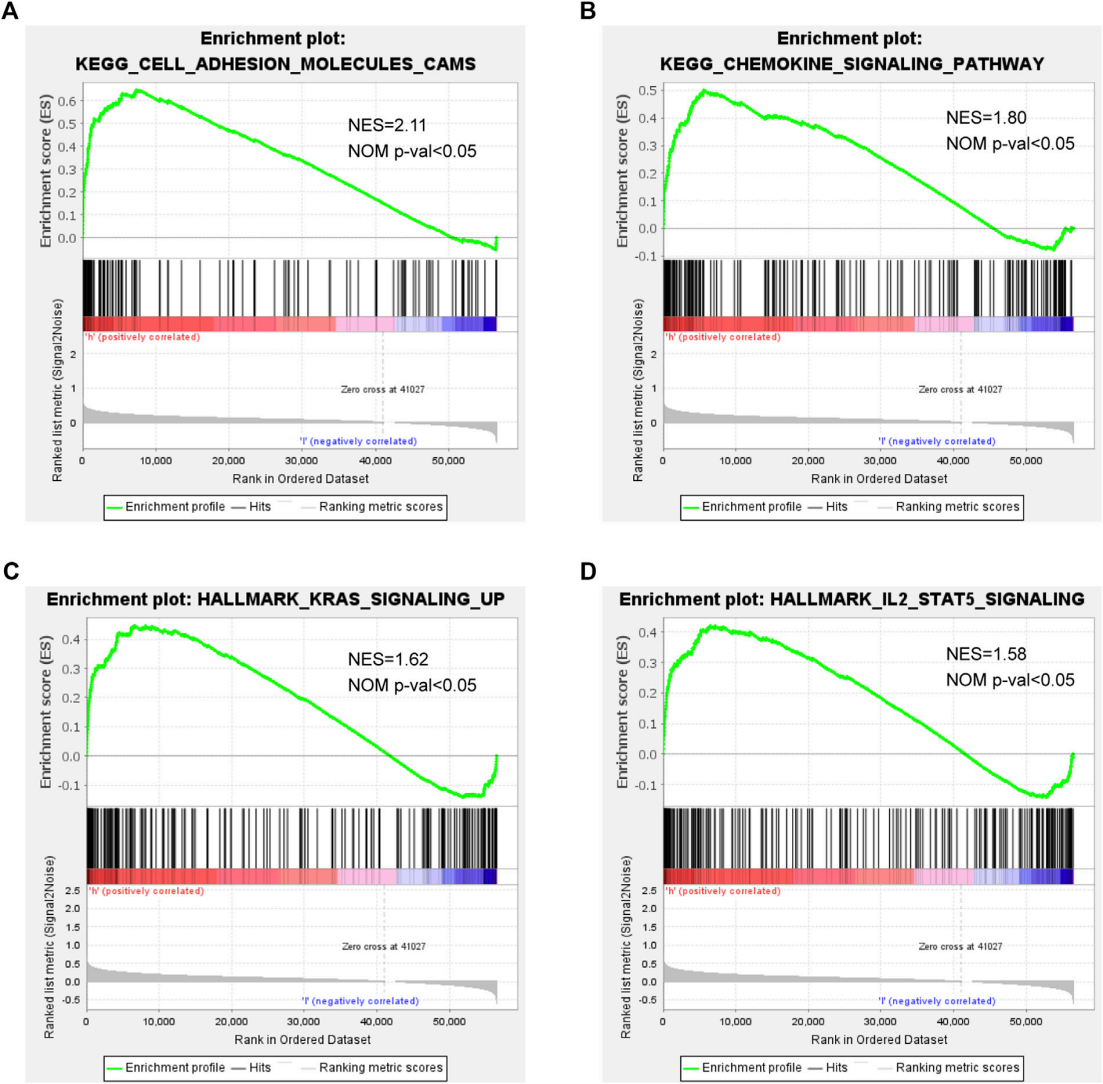
GSEA in high- and low-risk patients: **(A)** adhesion molecules, **(B)** chemokine signaling pathway, **(C)** KRAS signaling, and **(D)** IL-2/STAT5 signaling.

We also conducted GSEA for clarifying the possible biological functions and signal transduction pathways among high-risk patients. As shown in Figure 5, a higher risk score was correlated with adhesion molecules, chemokine signaling pathway, KRAS signaling, and IL-2/STAT5 signaling, indicating that the patients with these pathways might be more prone to a worse clinical outcome.

### Difference of Immune Checkpoints Between the High-Risk and Low-Risk Groups

**FIGURE 6:**
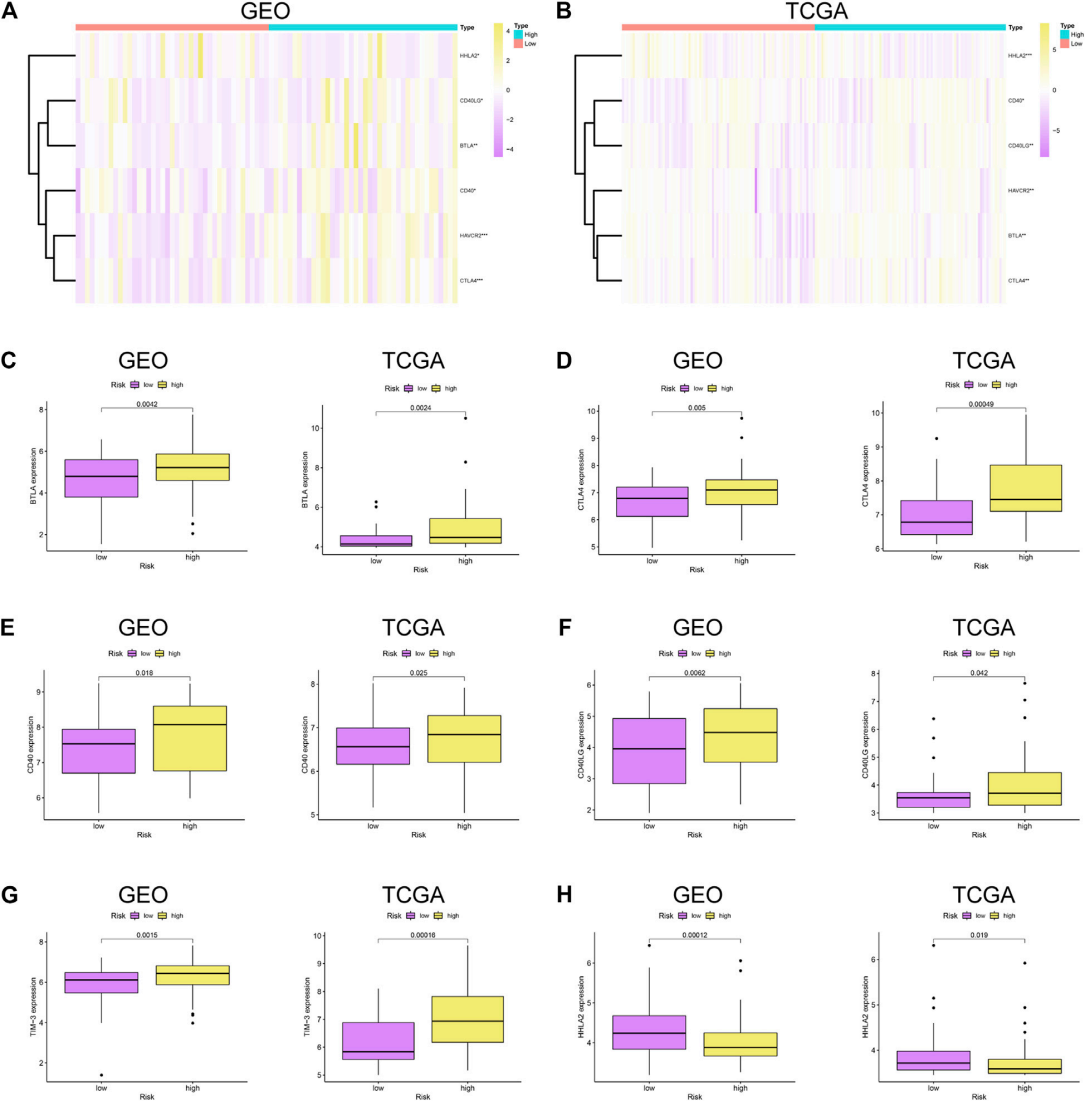
Immune checkpoint analysis. **(A–B)** Heatmap of immune checkpoints between high- and low-risk patients in GSE53625 and TCGA datasets. Differential expression of immune checkpoints in high- vs. low-risk patients: **(C)** BTLA, **(D)** CTLA4, **(E)** CD40, **(F)** CD40LG, **(G)** TIM-3, and **(H)** HHLA2.

To further explore the relationship between the immune checkpoints and two risk groups, we performed differentiation analysis for the expression of 22 immune checkpoints, including the TNF superfamily (BTLA, CD27, CD40LG, CD40, CD70, TNFRSF18, TNFRSF9, and TNFSF9) and B7-CD28 family (CD274, CD276, CTLA4, HHLA2, ICOS, ICOSLG, PDCD1, PDCD1LG2, and VTCN1), along with additional immune checkpoints (IDO1, HAVCR2, VSIR, LAG3, and NCR3). As shown in Figure 6, BTLA, CD40, CD40LG, CTLA4, and HAVCR2 were significantly upregulated in the high-risk group, while HHLA2 was enriched in the low-risk group.

### Validation of the Expression Patterns and Protein Expression of Prognostic Signature Genes

**FIGURE 7:**
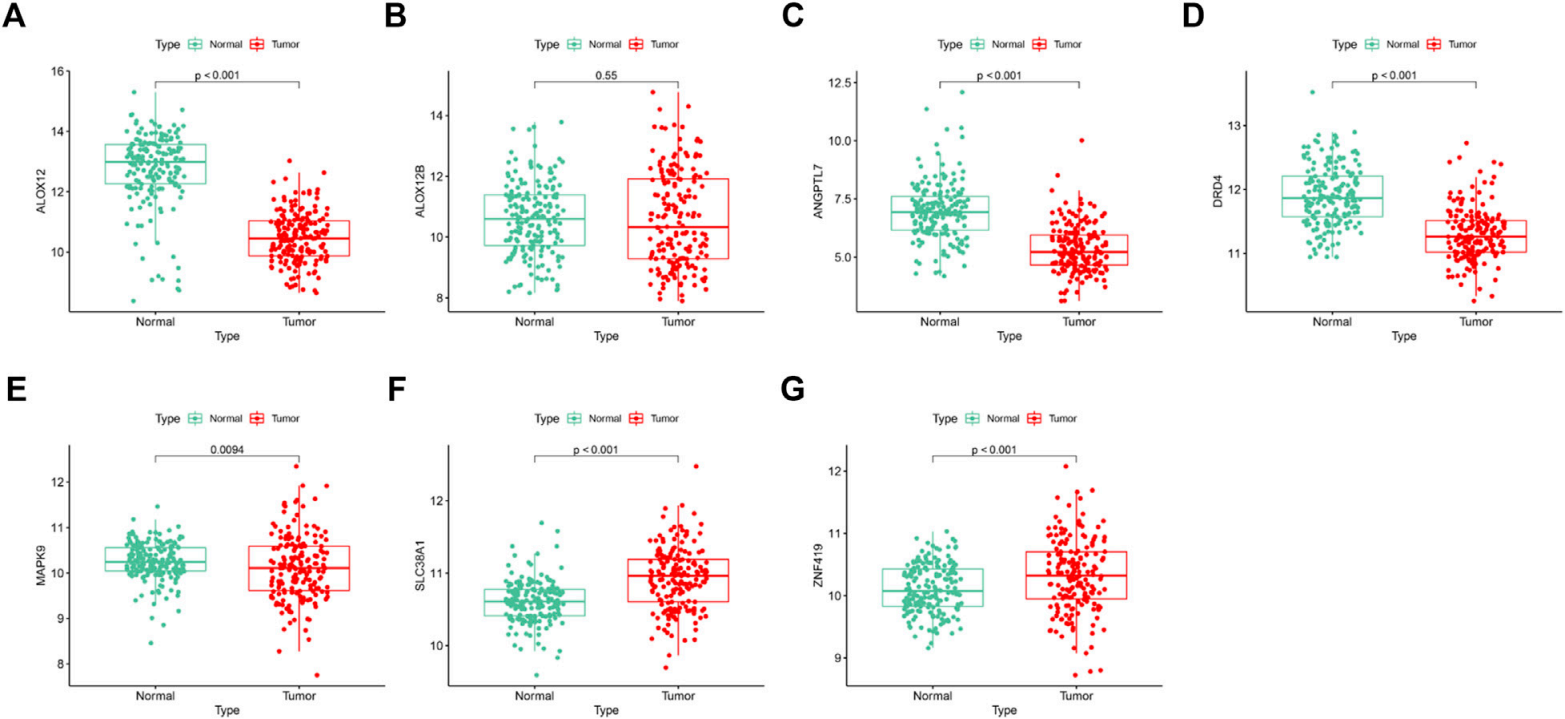
Differentiation analysis of the signature genes among ESCCs and normal tissues based on GSE53625 dataset.

**FIGURE 8:**
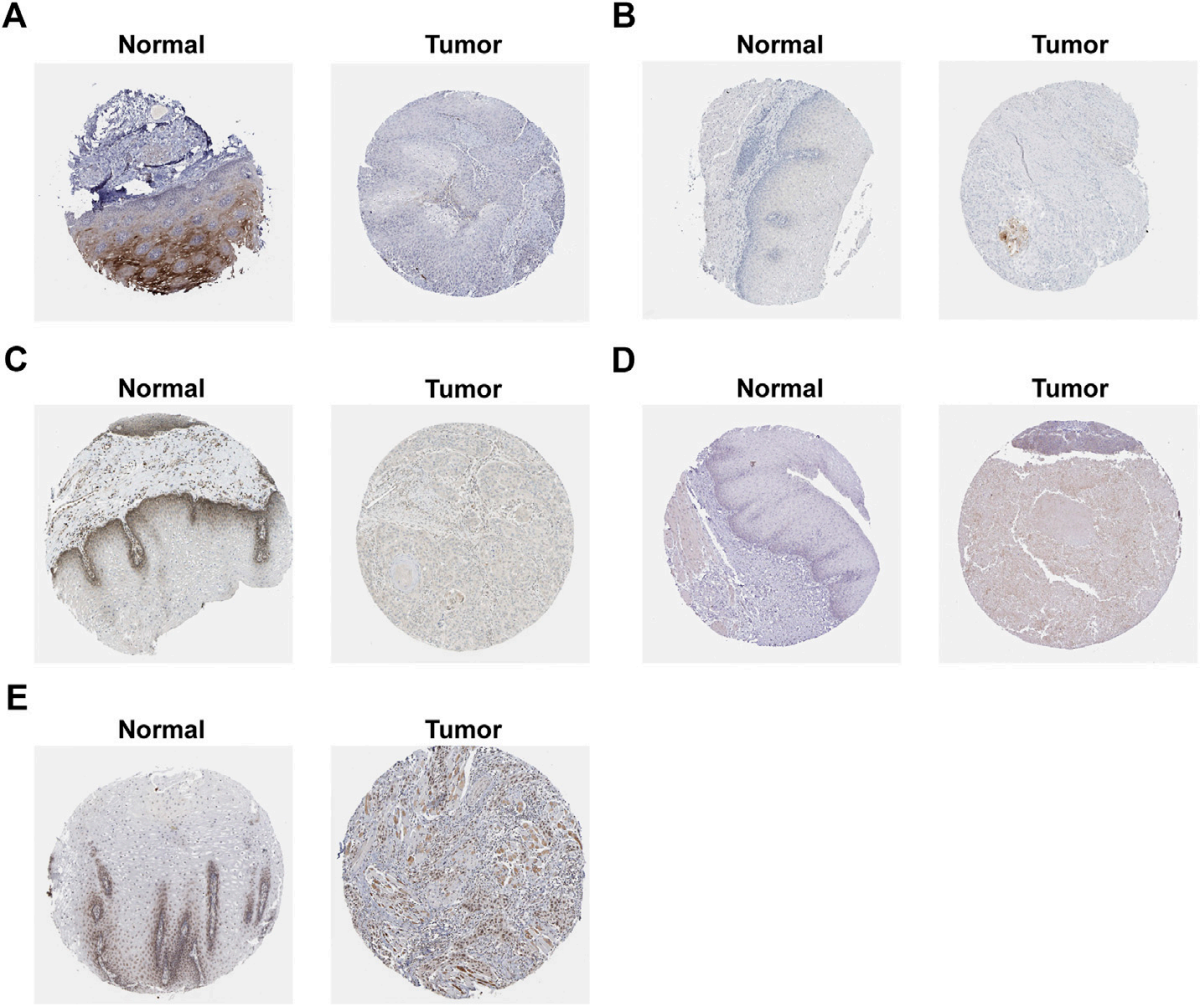
Protein expression of signature genes in HPA database. **(A)** protein expression of ALOX12. **(B)** protein expression of ALOX12B. **(C)** protein expression of MAPK9. **(D)** protein expression of SLC38A1. **(E)** protein expression of ZNF419.

We confirmed the expression levels of the seven signature genes (ALOX12, ALOX12B, ANGPTL7, DRD4, MAPK9, SLC38A1, and ZNF419) among the patients from GSE53625. The results showed that ALOX12, ANGPTL7, DRD4, and MAPK9 remarkably decreased within ESCC samples relative to non-carcinoma samples, whereas SLC38A1 and ZNF419 were highly expressed. Only ALOX12B expression showed no significant difference in tumor samples compared with normal samples (Figure 7). Consistent with the above results, the HPA database showed that ALOX12 and MAPK9 in ESCC tissues were lowly expressed, while SLC38A1 and ZNF419 were upregulated relative to normal samples. But DRD4 and ANGPTL7 protein expressions were not measured in the database (Figure 8).

### Inhibition of SLC38A1 Decreased Esophageal Squamous Cell Carcinoma Cell Proliferation and Migration

**FIGURE 9:**
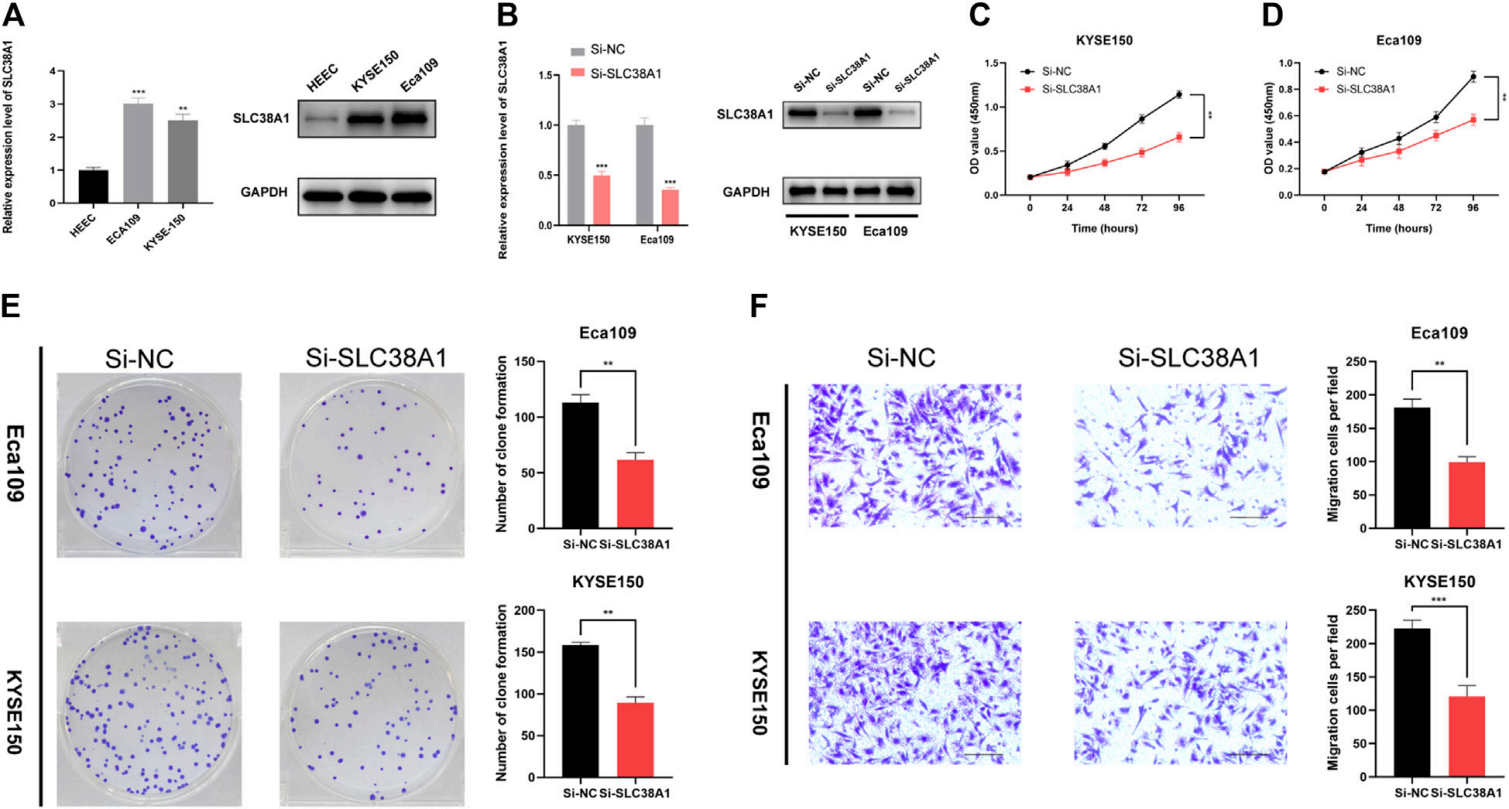
Effects of inhibiting the expression of SLC38A1 on ESCC cell proliferation and migration. **(A)** SLC38A1 was upregulated within KYSE-150 and Eca109 cells relative to HEECs by the qRT-PCR assay and western blot analysis. **(B)** The expression of SLC38A1 was downregulated in KYSE-150 and Eca109 cells by siRNAs. **(C–E)** KYSE-150 and Eca109 cell proliferation after anti-SLC38A1 siRNA transfection was measured using CCK-8 assays **(C, D)** and colony formation assays **(E)**. **(F)** Transwell assays for ESCC cell migration (scale bars: 100 μm).

Finally, we used the SLC38A1 gene to further explore the underlying role of our model in ESCC. First, the qRT-PCR assay and western blot analysis were performed to verify the differential expression between normal esophageal epithelial cells and ESCC cells (Figure 9A). As a result, SLC38A1 expression increased within ESCC cells relative to normal esophageal epithelial cells. Next, the siRNAs were applied to knock down the SLC38A1 levels within Eca109 and KYSE-150 cells, and both the qRT-PCR assay and western blot analysis confirmed the efficacy (Figure 9B). The CCK-8 proliferation assay and colony formation assay showed that downregulation of SLC38A1 can markedly reduce Eca109 and KYSE-150 cell proliferation (Figures 9C–E). Moreover, migration of Eca109 and KYSE-150 cells transfected with siRNA was inhibited (Figure 9F). These results suggest that SLC38A1 possibly promotes tumorigenesis of ESCC, yet the possible mechanism should be further explored.

## Discussion

ESCC is still a progressive and challenging disease with high morbidity and poor prognosis. Presently, the TNM staging system is still a crucial prognostic factor for assessing the prognosis of cancer patients, but it has limitation in elucidation of genetic variations, and those at the identical stage present a powerful heterogeneity for prognostic outcome. Selectively inducing the death of cancer cells may account for an efficient way to treat cancer. Mounting evidence indicated that ferroptosis plays a significant role in tumorigenesis and treatment for cancer (Yang et al., 2014; Stockwell et al., 2017; Liu et al., 2018; Carbone and Melino, 2019; Hassannia et al., 2019; Liang et al., 2019; Tesfay et al., 2019). However, there has not yet been much systematic analysis in the context of ferroptosis in ESCC, and the underlying mechanism of ESCC remains poorly illustrated.

This work concentrated on ferroptosis-related gene signatures with the prognosis value of ESCC patients. In the GEO training set, we first identified prognostic ferroptosis-related genes and then built the predictive model comprising seven FRGs through integration of LASSO regression and Cox regression analysis. According to Kaplan–Meier curve analysis, high-risk cases were associated with dismal OS compared with low-risk counterparts. Meanwhile, the ROC curve illustrated good performance of our model. The AUCs of ROC plots for five-year OS in the GEO cohort and TCGA cohort were 0.788 and 0.848, respectively. Furthermore, ROC curves were utilized to compare the prediction capability of our proposed model with that of other signatures. As a result, our risk signature achieved consistently excellent predictive value, compared with other published risk prognostic signatures in ESCC (Wang et al., 2020b; Gao et al., 2021; Zhao et al., 2021). The constructed prognostic signature was also verified in the GEO test set, entire GEO set, and TCGA set. Next, the seven ferroptosis-related genes’ signature predicted the dismal OS for ESCC cases after subgroup analysis according to age, gender, clinical stage, T stage, and N stage. The results of Cox regression analysis showed that the as-constructed risk model might serve as an independent risk factor for ESCC. Moreover, the nomogram was established and the calibration plots were used to examine whether our nomogram was accurate in the prediction of one-, three-, and five-year OS. All these results revealed that the ferroptosis-related signature could be a superior predictor compared with the traditional clinical indicator.

Our proposed ferroptotic signature was composed of seven ferroptosis-related genes (ALOX12, ALOX12B, ANGPTL7, DRD4, MAPK9, SLC38A1, and ZNF419). Among the seven genes, ANGPTL7, MAPK9, and ZNF419 are latent hazardous genes and ALOX12, ALOX12B, DRD4, and SLC38A1 are potential protective genes. All these genes were shown to participate in the initiation and development of various cancers. ALOX12 belongs to a family of lipoxygenases (LOXs) with a reported role in the promotion of the oxidation activity of polyunsaturated fatty acids (Yoshimoto et al., 1992). The ALOX12 protein could foster the biosynthesis of 12-hydroxyeicosatetraenoic acid by specifically metabolizing arachidonic acid (Honn et al., 1994). It has been confirmed that ALOX12 has the capability of mediating inflammation, cell migration, apoptosis, and tumor cell proliferation (Zheng et al., 2020). Yang et al. found that ALOX12 was downregulated in recurrence of hepatocellular carcinoma and regulated the ALOX12–12HETE–GPR31 signaling pathway (Yang et al., 2019). In lung cancer, overexpression of ALOX12 facilitated cell growth and migration by promoting RhoA and NF-κB activity (Chen et al., 2020). ALOX12B protein, another isoform of arachidonic acid 12-lipoxygenase, mainly catalyzes arachidonic acid to 12R-hydroxyeicosatetraenoic acid (Zheng et al., 2011). Jiang et al. revealed that the inhibition of ALOX12B could restrain cervical cancer cell proliferation and growth through suppressing the PI3K/ERK1 pathway, suggesting that it can be taken as a good biomarker to provide new therapeutic strategies for cervical cancer patients (Jiang et al., 2020). In addition, Chu et al. reported that ALOX12 could oxygenate polyunsaturated fatty acids, which in turn induce p53-mediated tumor cell ferroptosis (Chu et al., 2019). Consistent with previous studies, our results indicate a negative correlation between ALOX12 and the poor prognosis of patients.

ANGPTL7, a member of the angiopoietin-like protein (ANGPTL) family, consists of an N-terminal coiled-coil domain and a C-terminal fibrinogen-like domain. The same structural domain as angiopoietin ensures ANGPTL7 to promote angiogenesis (Carbone et al., 2018). For instance, Parri et al. gave us a hint that hypoxia induced ANGPTL7 expression in tumor cells, which exert a vital part in pro-angiogenetic development (Parri et al., 2014). It was reported that ferroptosis induced by erastin or RSL3 could downregulate ANGPTL7, which might be involved in the onset of ferroptosis in cancer cells (Yang et al., 2014). The higher expression level of ANGPTL7 was also observed in colorectal cancer based on the gene profile analysis (Liu and Zhang, 2017). Our results are in line with these research studies, pointing out that ANGPTL7 is a risky gene (HR > 1) in ESCC. The DRD4 gene encodes the G-protein–coupled receptor which could suppress the activity of adenylyl cyclase. In glioblastoma, DRD4 could promote proliferation and autophagic flux and enhance survival of glioblastoma stem cells (Dolma et al., 2016). Wang et al. demonstrated that ferroptotic erastin contributed to degradation of DRD4 protein and anti-ferroptotic dopamine impeded DRD4 protein declination (Wang et al., 2016). MAPK9 could phosphorylate a series of transcription factors, which subsequently regulates cell proliferation, migration, and programmed cell death. Li et al. discovered that MEG3 and MIAT may foster the progression of lung adenocarcinoma through interacting with miR-106, thus regulating the involvement of MAPK9 in the MAPK signal transduction pathways (Li et al., 2016). SLC38A1, also known as amino acid transporter system A1, was initially identified as a crucial transporter of glutamine (Gu et al., 2001). SLC38A1 has been proved to be a potential oncogene in colorectal cancer and gastric cancer (Xie et al., 2014; Zhou et al., 2017). As a transcriptional regulator, ZNF419 polymorphism at the splice donor site might result in novel minor histocompatibility antigen ZAPHIR related to renal cell carcinoma (Broen et al., 2011).

Immune checkpoints could exert tumor immunosuppressive effects, which in turn prevent tumors from immune attack. BTLA was a member of the TNF superfamily, and its expression was associated with cancer aggressiveness (Wang et al., 2020c). TIM-3, also known as HAVCR2, was predominantly located on NK cells and macrophages, inhibiting the activation of anti-tumor immunity (Datar et al., 2019). Matsumura et al. indicated that CD40 expression in ESCC is closely correlated with tumorigenesis and lymph node metastasis (Matsumura et al., 2016). In our results, most of immune checkpoints were related to the high-risk group, which verify the reliability of the signature in evaluating the prognosis of patients. Notably, some of the signature genes also have intricate connection with immune checkpoints. For example, MAPK9, also known as JNK2, was confirmed to be involved in the regulation of B7.1 (CD80) which could interact with CTLA-4 to mediate the development of immune responses. Lim et al. found that the expression of B7.1 induced by LPS was significantly suppressed by siJNK2 RNAs (Lim et al., 2005). It is reasonable to speculate that the downregulation of MAPK9 in ESCC might facilitate carcinogenesis through inhibiting B7.1-mediated activation of immune responses. In addition, restriction of glutamine utilization could enhance anti-programmed death ligand-1 (PD-L1) levels in tumor, which promote the effectiveness of PD-L1 antibody (Byun et al., 2020). Therefore, we hypothesized that SLC38A1, a key transporter of glutamine, might block the effectiveness of PD-L1 antibody by stimulating glutamine metabolism in ESCC.

Finally, we sought to detect the relationship between SLC38A1 and ESCC progression. The results showed that inhibiting SLC38A1 suppressed the cell viability and migration of Eca109 and KYSE-150 cells, which further proved the carcinogenic role of SLC38A1 in digestive-system neoplasms.

There are several limitations of this study. First, the data analyzed in the present work might be acquired from the public database. The clinical effectiveness and credibility of the as-constructed signature should be further verified by more practical data. Second, the functional mechanisms of signature need to be explicated through more profound *in vivo* and *in vitro* experiments.

To sum up, this work first identifies a new FRG-based prognostic signature, which predicts the OS of ESCC and mirrors the immune status. This constructed signature will provide new options for individualized treatment.

## Data Availability

Publicly available datasets were analyzed in this study. These data can be found here: TCGA (https://portal.gdc.cancer.gov/), GEO database (https://www.ncbi.nlm.nih.gov/geo/), FerrDb website (http://www.zhounan.org/ferrdb) and HPA database (https://www.proteinatlas.org/).
